# What do we need to address when we treat neglected Monteggia fracture in children

**DOI:** 10.3389/fped.2024.1430549

**Published:** 2024-08-29

**Authors:** Yangfei Yi, Can Liu, Zheng Xu, Yuyin Xie, Shu Cao, Jie Wen, Xiaohong Jian, Yufei Li

**Affiliations:** ^1^ Department of Anatomy, Hunan Normal University School of Medicine, Changsha, Hunan, China; ^2^ Department of Pediatric Orthopedics, Hunan Provincial People’s Hospital, The First Affiliated Hospital of Hunan Normal University, Changsha, China

**Keywords:** neglected Monteggia fracture, length ratio of the ulna and radius, proximal ends of the ulna and radius, annular ligament, proximal radioulnar joint

## Abstract

Monteggia fracture is a relatively uncommon injury in pediatric patients, accounting for less than 2% of forearm fractures, characterized by a combination of ulna fracture and radial head dislocation. Neglected Monteggia fractures define as those that have not received treatment within 3 weeks. In children, ulna fractures are easily diagnosed while radial head dislocation may be overlooked, necessitating open reduction after neglecting the Monteggia fracture and potentially causing additional trauma to the child. This study aims to review the pathological characteristics of neglected Monteggia fractures based on the length ratio of the ulna and radius, relative positions between the proximal ends of the ulna and radius, the integrality of annular ligament and the pathological change of proximal radioulnar joint. The findings will provide valuable insights and guidance for managing neglected Monteggia fractures.

## 1 Introduction

The incidence of upper extremity fractures in children's fracture ranges from 20% to 30%. Monteggia fracture, a type of forearm fracture in children, refers to the complex injury involving any part of the ulna combined with multi-directional dislocation or radial head fracture (1). It is a relatively uncommon type of forearm fracture, accounting for less than 2% of all pediatric forearm fractures (2). If acute Monteggia fracture in children is not appropriately treated and persists for more than 4 weeks, it may progress into neglected Monteggia fracture (NMF) (3), leading to various clinical symptoms such as elbow pain, restricted range of motion, increased valgus deformity, and nerve entrapment issues (4). In neglected Monteggia fractures, after dislocation of the radial head from the annular ligament, the ligament that originally protected the radial head becomes entrapped and incarcerated outside the humeroradial joint (5). After a prolonged duration, an extensive accumulation of scar tissue within the joint capsule will result in its enlargement, leading to contracture and calcification of ligaments and joint capsules, thereby further impeding radial head reduction (6). Moreover, excessive growth and hypertrophy of the radial head along with thinning of the radial neck will exacerbate difficulties in achieving radial head reduction. Concurrently, some patients may develop proximal ulnar heterotopic ossification, causing shallowing or even disappearance of the ulnar radial notch. These series of pathological changes not only impact the aesthetic appearance and limb function but also hinder psychological development in children by limiting their engagement in social activities and affecting mental well-being (7).

Currently, numerous scholars have conducted extensive research on the treatment of Monteggia fractures in children. The primary intervention methods for NMF include ulnar osteotomy combined with plate internal fixation or an extendable external fixator (8). Previous follow-up studies have revealed that some patients still experience varying degrees of forearm rotation function loss after surgery (9), while others suffer from limited elbow flexion due to re-dislocation (10). Additionally, long-term joint dislocation and impingement can lead to osteoarthritis in certain patients. In order to achieve more precise treatment and reduce postoperative complications, researchers have begun focusing on the pathophysiological pathogenesis of neglected Monteggia fractures in children, exploring whether annular ligament repair or reconstruction is necessary, as well as investigating different ulnar osteotomy points and prebending angles of plates (11). At present, some scholars are dedicated to investigating human anatomy and imaging perspectives (12), as well as guiding surgeons in restoring various structures to their normal state (13) during operations based on the growth and developmental patterns of a healthy child's forearm. This approach aims to achieve functional outcomes close to normal and minimize complications. Neglected Monteggia fractures often result in ulnar angulation deformity, ulna/radius imbalance, annular ligament incarceration, and dislocation of the upper radioulnar joint. Although different clinical treatment methods have shown certain therapeutic effects, there is still a lack of standardized treatment protocols and long-term follow-up studies.

**Figure 1:**
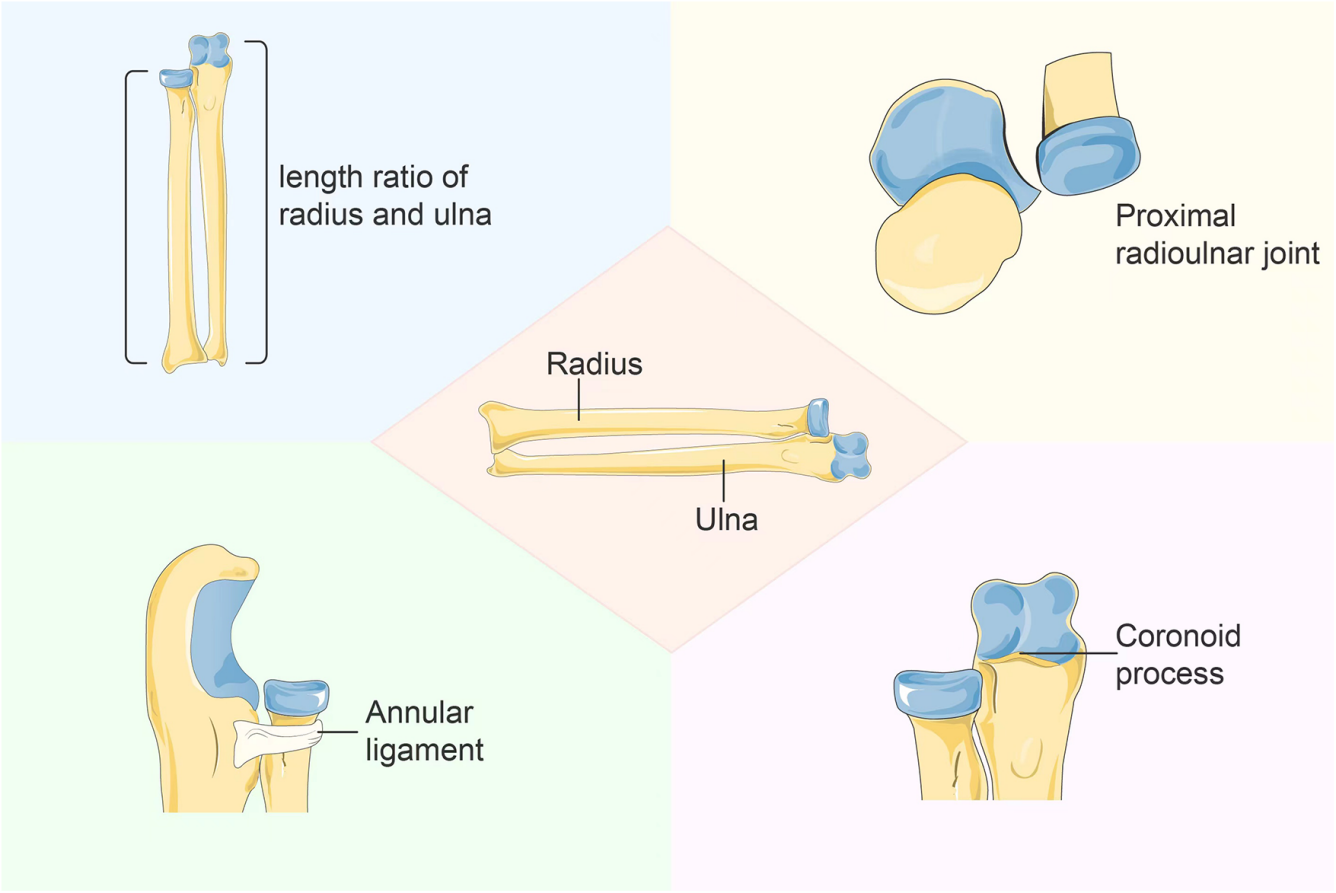
Pathological changes of neglected Monteggia fracture.

Considering the pathological characteristics of Neglected Monteggia fractures, this review aims to discuss key considerations when treating neglected Monteggia fractures in children, including the length ratio between the ulna and radius, relative positions of their proximal ends, integrity of the annular ligament, and pathological changes in the proximal radioulnar joint. Additionally, we aim to explore various treatment strategies for neglected Monteggia fractures with dislocation and evaluate their effectiveness to provide valuable insights for researchers (Figure 1).

## 2 Length ratio between the ulna and radius

The ulna and radius are the two primary long tubular bones that constitute the human forearm, forming its structural support and facilitating precise hand movements and power output (11). The ulna is positioned medially in the forearm, while the radius is located laterally. These bones articulate with other bones at the elbow and wrist joints, creating a complex joint structure (14) that enables flexible rotation and grasping actions of the forearm. Numerous anatomical experiments have demonstrated their involvement in various upper limb joints such as brachioulnar, upper radioulnar, lower radioulnar, and radiocarpal joints; moreover, during pronation and supination of the forearm, the radius rotates around the ulna (15). Consequently, changes in ulnoradial length have been identified as an independent risk factor for radial head dislocation (16).

Long-term dislocation of the radius can result in proximal bone deformity, and even after reduction, there is a risk of secondary dislocation due to mismatch between the humeral surface and articular surface (17, 18). Currently, ulna osteotomy with angulation has become the mainstream surgical approach for older patients and refractory Neglected Monteggia fractures (19). Some scholars argue that ulna osteotomy plays a crucial role in annular ligament reconstruction because it allows for posterior movement of the ulnar angle (14). By pulling the radius through the interosseous membrane, radial head reduction is achieved while adjusting the ulnar angle to maintain stability. Studies have demonstrated that younger age and shorter duration of dislocation increase the likelihood of successful surgery (20).

Oka et al. (20) discovered that in cases of Neglected Monteggia fracture occurring within 3 years after the initial injury, there was no significant disparity in the length of ulna and radius between the affected and healthy sides. However, for patients with disease onset exceeding 3 years, the length of radius on the affected side resembled that on the healthy side, while the ulna on the affected side exhibited a significantly shorter length compared to its counterpart on the healthy side. Some researchers propose lengthening of ulna in children as a preventive measure against ulnar variation and recurrence of radial head dislocation. However, the precise extent of ulna lengthening remains uncertain, while some patients have experienced wrist and elbow impingement. Furthermore, there is currently no standardized criterion for determining the length of ulna lengthening. Ning et al. (21) discovered a positive correlation between age and both radius and ulna in consistent proportions. In individuals with short-length type, the anterior ratio of radius to ulna (RLRU) ranged from 0.8941 to 0.9251, while the lateral ratio ranged from 0.8936 to 0.9375. For those with full-length type including epiphyseal length, the anteroposterior ratio of radius to ulna length was found to be between 0.9286 and 0.9508, whereas the lateral ratio ranged from 0.9579 to 0 (22, 23).

**Figure 2:**
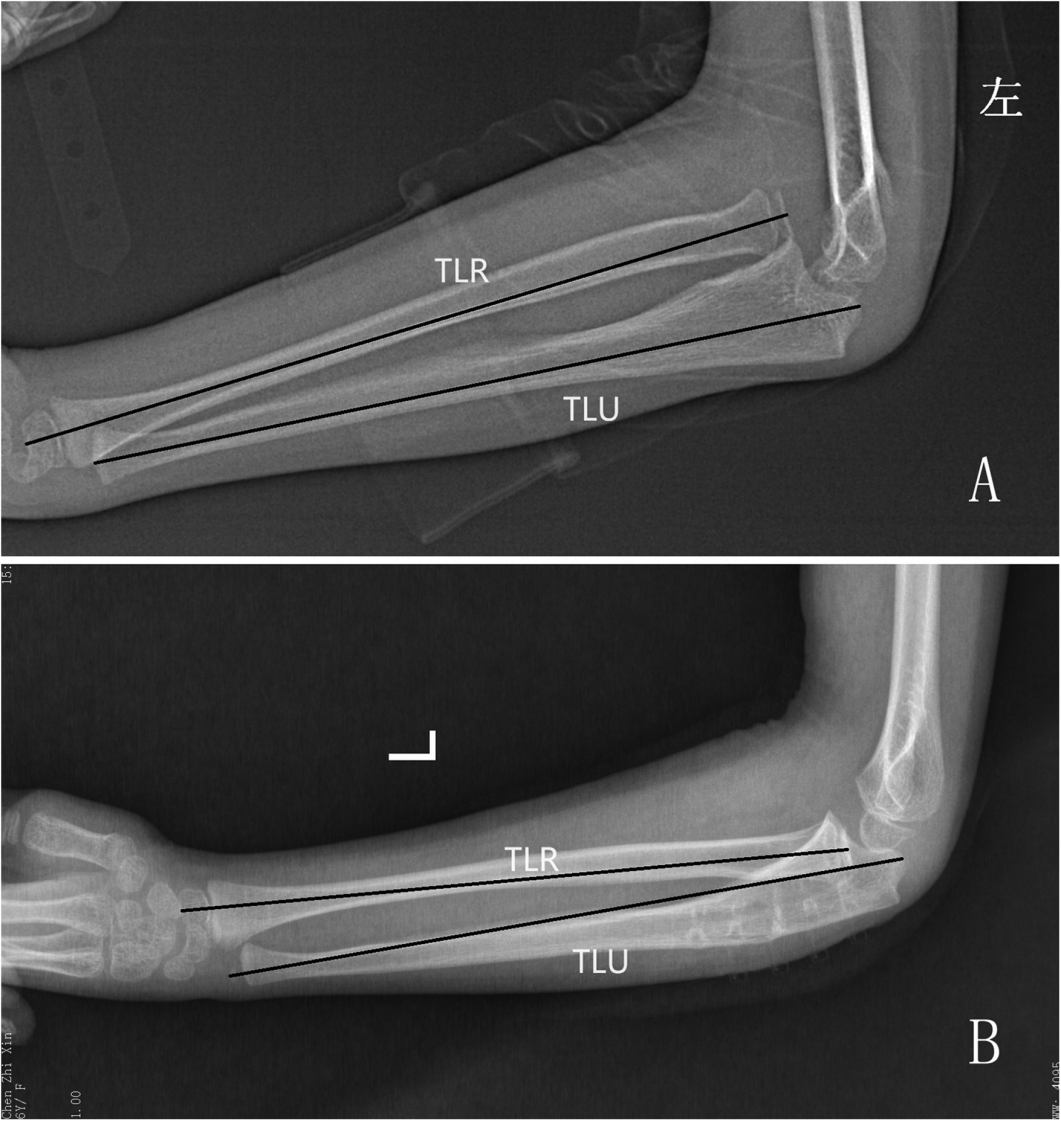
X-rays of neglected Monteggia fracture children, length ratio between the ulna and radius improved after operation. **(A)** Pre-operation, **(B)** Post-operation, TLU, total length of ulna; TLR, total length of radial.

Although the measurement data are highly accurate, their utilization in the management of neglected Monteggia fractures has been limited, and their clinical value and guidance remain uncertain. In the diagnosis and treatment of pediatric cases with neglected Monteggia fractures, particular attention should be devoted to restoring or preserving the radial arch and ensuring the integrity of the interosseous membrane (Figure 2). Failure to restore the radial arch within 5% deviation from that of the contralateral forearm may result in a loss exceeding 20% in forearm rotation function (24).

Additionally, residual angulation deformity can lead to restricted rotation; hence, ulnar length recovery may serve as an adjunctive measure for radial head reduction. The degree of looseness of the radial head correlates with the degree of ulnar-radial deformity, and children whose osteotomy point is closer to the end have a significantly better prognosis and a lower complication rate. However, the choice of ulna osteotomy site remains a subject of controversy. Some scholars have suggested performing the osteotomy at 1/4 of the proximal ulna (25), as this location allows for increased space to accommodate radial head reduction and prevents subsequent radial head prolapse, provided that sufficient length is maintained for proximal plate fixation (26).

Alternatively, other scholars propose performing the osteotomy at the most prominent angle deformity of the ulna. This approach not only resolves irreducible radial head issues but also maximizes correction of ulnar deformities. Nevertheless, no definitive research data currently exist to guide decision-making regarding how long exactly on the ulna to perform an osteotomy. Imbalances between the radius and ulna may give rise to additional complications such as wrist pain, joint impingement, or limited joint function (27). Accurate proportional data considering both age-related increases in radius and ulnar length are crucial for achieving optimal recovery with ideal correction of both aesthetic deformities and functional restoration.

## 3 Relative positions between the proximal ends of the ulna and radius coronoid process

The coronoid process of the ulna serves as a proximal extension of the ulnar diaphysis, providing resistance against the forces exerted by the biceps, brachialis, and triceps brachii muscles during flexion and extension (28). Moreover, it plays a crucial role in maintaining axial stability of the elbow joint and ensuring stability during posteromedial and posterolateral rotation. Additionally, it acts as a static constraint to prevent varus or valgus deformities of the elbow joint while also restraining posterior translation and rotation (29). Biomechanical studies have emphasized its critical function in supporting the elbow joint to prevent dislocation (30). Radial head dislocation and a shorter proportional ulnar length were independent risk factors associated with diminished forearm rotation, supination and pronation together, and in conjunction with other risk factors, could be used to predict range of motion (31). Morphological alterations in the radial head can lead to abnormal development of the ulna, resulting not only in acute or chronic joint instability (32) but also posterior or recurrent dislocations of the elbow (33).

Some studies has been found that when the radial head is in the same plane as the coronoid process of the ulna on lateral x-ray view, it affects the effective restoration of ulna length. When the radial head was at the same level as the coronoid process on lateral radiographs, the ulna was gradually pulled away to allow the ulna to continue lengthening, and the radius was slightly below the plane of the coronoid process, which allowed the radial head to be reset normally and the distal ulnar-radial joint to normalize. Among the follow-up patients, no patient developed radial head dislocation, and the function and appearance of the upper limbs were significantly improved (34). Huang et al. found that even when the radial head dislocation could not be reduced, the elbow pseudovarus deformity could be well treated by pulling the radial head down to the plane of the coronoid process, so as to improve the elbow function. All cases obtained satisfactory appearance of the elbow and good functional recovery of the forearm (35).

Neglected Monteggia fracture is caused by missed diagnosis or delayed treatment (36). After a long time of injury, the morphological changes of the radial head occur, and the normal humeroradial joint alignment is lost (37). The humeroradial joint consists of the humeral tuberosity (convex) and the radial tuberosity (concave), which together with the ulnar joint form a composite synovial joint, the elbow joint, which is also a uniaxial joint. The patient's radial head was enlarged and widened, and beyond the coronoid process on the lateral x-ray film. Even if the proximal radioulnar joint was reluctantly reduced, it was difficult to maintain long-term stability (38). In addition, the morphological change of the radial ulnar notch is also a risk factor for postoperative re-dislocation of neglected Monteggia fracture in children. Patients with convex radial ulnar notch morphology have a high incidence of re-dislocation and poor elbow function after operation (39).

When the morphological changes of radial head dislocation occur in neglected Monteggia fracture, the common treatment options include non-surgical treatment and surgical treatment (40). Most patients with morphological changes of the radial head need surgical treatment (41). Surgical treatment includes open reduction of the radius and lengthening of the ulna, which can quickly restore the dislocation while avoiding long-term adverse effects. Although the lengthening of the ulna and open reduction of the radius have been proved to be effective in most clinical cases, the positional relationship between the radial head and the coronoid process is particularly important, which not only affects the recovery of the length of the ulna, but also affects the late stability of the elbow joint (42–44). At present, the position relationship between the radial head and the coronoid process is still controversial. Whether it is proposed that the radial head should be reduced to the same plane as the coronoid process or the radial head should be slightly lower than the coronoid process, there are not enough clinical cases and long-term follow-up to confirm its view. In any case, it is undeniable that restoring the proximal radius and coronoid to a normal anatomical positional relationship can stabilize the flexion of the elbow (45–47).

## 4 Integrality of annular ligament

Annular ligaments are ligaments located around the annular articular surface of the radius and proximal ulna, the annular ligament is a part of the collateral ligament that encircles the radial head and gradually reduces to the distal radial neck, connecting and stabilizing the radioulnar joint during the whole supination and pronation (41). Galik et al. (48) found that the annular ligament injury increased the anteroposterior and medial and lateral movements of the radial head by 44% and 24%, respectively. This suggests that the annular ligament serves to stabilize the radial head. The shape of the annular ligament can change (49). When Monteggia fracture occurs, the instability of the joint may lead to the injury or rupture of the annular ligament (50). Some researchers have proposed three pathological changes of the annular ligament associated with neglected Monteggia fracture injury (51). First, ligaments may tear and remain ruptured. Second, the ligament was torn and compressed posteriorly to the radial head, thereby preventing its reduction. Third, the radial head dislocated from below the ligament and was located in front of the annular ligament (52). Sandman et al., (53, 54) found that dislocation of the proximal ulna combined with annular ligament tears can affect elbow biomechanics and may lead to subluxation of the radial head. An old injury to the annular ligament means that some time has passed since the fracture and the ligament injury has healed or scar tissue has formed. This condition leads to joint instability and impaired function. However, some recent studies have reported that all annular ligaments in neglected Monteggia fractures are intact and displaced and can be relocated and used. Pesl believes that the annular ligament is a unique anatomical structure that maintains the position of the radial head, and reconstruction of the annular ligament minimizes the chance of displacement of the radial head and guarantees the stability of the radial head during mobilization.

On the other hand, Sandman et al. believed that the effect of annular ligament reconstruction was not very ideal (55). After fracture, the annular ligament had adhered to the scar, and the reconstruction was too complicated. Moreover, the reconstructed ligament could not grow with the development of the radial neck (56), which in turn limited the development of the radial neck, and the rotational movement of the radial head and neck was limited after reconstruction (57). At the same time, they believed that the ulna osteotomy orthopedic surgery was sufficient to provide sufficient stabilizing force, so there was no need to repair or reconstruct the annular ligament (58). The annular ligament is located at the base of the radial head and neck, it is located around the circumferential articular surface of the radius and attaches at both ends to the anterior and posterior margins of the ulnar radial notch, which, together with the ulnar radial notch, forms a fibro-osseous ring that accommodates the radial head (59). According to the analysis of lever mechanics principle, the annular ligament has the longest moment, which is the most effective factor to stabilize the radial head and prevent lateral dislocation. However, the radius of the radial head is very small, and its rotational limiting moment is also very small. We believe that the tightness and other aspects of the reconstructed annular ligament may be different from those before injury, and surgery should be performed according to specific circumstances (60). However, if the residual annular ligament is sufficiently long, it should be reconstructed to approximate biological characteristics by disconnecting and utilizing the original annular ligament as much as possible. If the annular ligament is healed with the scar and cannot be dissected (61), the annular ligament is reconstructed by expanding the fibrous scar as a whole or by taking a deep fascia from the dorsal forearm (62). As the dislocated radial tuberosity loses the restriction of the humeral tuberosity and overgrows, the radial tuberosity and neck become enlarged, and the reconstructed annular ligaments around its neck are laxer than normal ligaments, and the radial head does not overgrow after repositioning. After reduction, the radial head will not overgrow (63), and the reconstructed ligament may have no significant effect on the development of the radial neck (64). The treatment of neglected Monteggia fracture with annular ligament injury should be individualized according to the specific situation. Repair or reconstruction of the annular ligament may be required at the time of surgery to provide stable joint function. The rehabilitation phase is also very important, and proper rehabilitation and functional exercises help to restore joint function and strength.

## 5 Pathological change of proximal radioulnar joint

**Figure 3:**
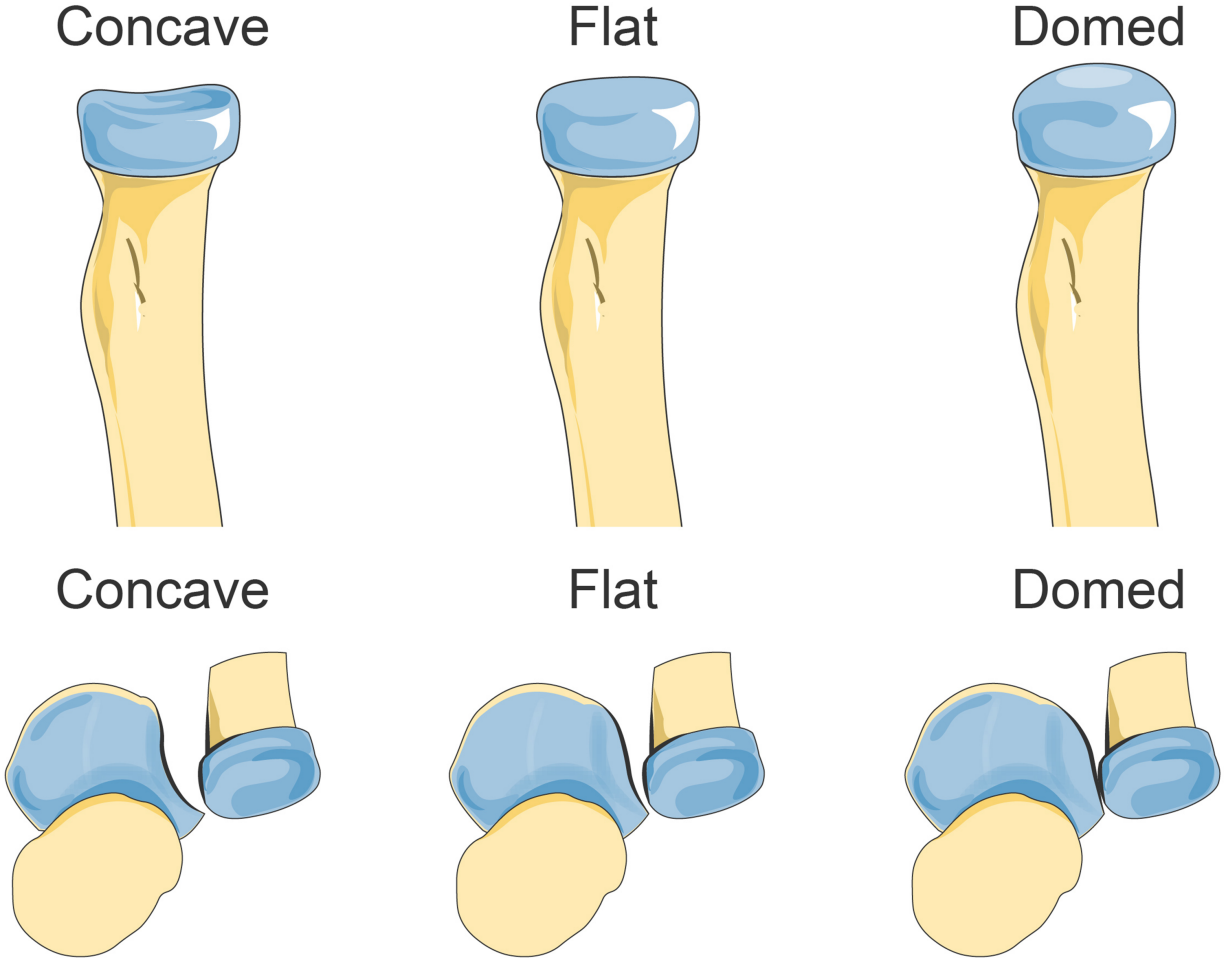
The different morphologic features of the radial head and radial notch. Reprinted with permission from “Missed Monteggia fractures in children treated by open reduction of the radial head and corrective osteotomy of the ulna.Sci Rep 12, 16819 (2022).” by Shu Cao, Zhong-Gen Dong, Li-Hong Liu, Jian-Wei Wei, Zhao-Biao Luo & Ping Peng, licensed under Creative Commons CC BY, Springer Nature. Copyright ©The Author(s) 2022. Published by Springer Nature, https://www.nature.com/articles/s41598-022-21019-4.

The proximal radioulnar joint is an axle joint with a cylindrical surface, resembling a ball bearing system (65). The annular ligament encloses the ball bearing system and extends from the anterior edge to the posterior edge of the radial notch of the ulna (66). Although slightly oval in shape, the radial head exhibits concavity. During pronation, it laterally shifts by approximately half of the difference in its two axes’ lengths, allowing for movement of the radial tuberosity into the supinator fossa (the attachment site for the supinator muscle) on the ulna. Neglected Monteggia fracture results in long-term deformity and dislocation of both ulna and radius (67–69). Prolonged dislocation causes enlargement and deformation of proximal radius, leading to loss of its original “concave disc” shape which does not entirely align with that of ulnar radial notch (Figure 3). The posterior inclination angle of the concave articular surface of the radial head increases during forearm supination. The longitudinal axis of the concave articular surface aligns with the rotational axis of the forearm, establishing a close anatomical connection with the humeral joint. During forearm pronation, there is a tilt towards the radial side in the radial head concave joint. Several studies have demonstrated that when both the radial head and radial notch exhibit a concave appearance, there is a lower rate of subluxation/redislocation compared to cases where they are flat or round (5.56% vs. 81.82%, respectively) (70). Neglected Monteggia fractures leading to persistent dislocation of the radial head can result in elbow pain, neurological deficits, reduced range of motion, and valgus deformity.

The interval time is a crucial factor that influences the morphology of the radial head. Consequently, a longer duration between injury and surgery leads to more severe morphological abnormalities in the radial head and increases the likelihood of postoperative redislocation (71). In patients with an interval of less than 6 months between injury and open reduction, no significant changes were observed in the radial head. Lu (72) noted that intervals longer than 6 months tend to result in increased growth or overgrowth of the radial head. This suggests a direct correlation between pronounced proliferative changes at the proximal ulna and subluxation/relocation of the radial head since excessive growth hinders proper anatomical positioning due to incongruity with radial head deformity, while proliferative changes in the radial notch impede correct alignment (73). Another study found that approximately 62.5% of dislocated patients exhibited a larger head-neck ratio on the affected side compared to their normal side, while 41.67% had irregularly shaped radial heads, and 41.67% showed at least a 5° difference in cervical axis angle compared to their unaffected side (74–76).

## 6 Treatment

Studies have reported successful surgical correction of dislocated joints after 6 years (77). Proximal ulna lengthening has been shown to restore joint coherence even several years after injury (78). Nakamura et al. (79) performed open reduction, ulna osteotomy, and annular ligament reconstruction in pediatric cases with good long-term clinical results expected if surgery was performed before the age of 20 or within 3 years of injury (80, 81). Early radial head resection may relieve pain and deformity but does not improve movement due to soft tissue contractures that form (78–80). Excessive angular deformity of the proximal radius can lead to subluxation during rotation (81). The main complications of radial head resection are proximal displacement of the radius and positive variation of the ulna.

The majority of radial bones exhibit a proximal displacement of 2 to 3 mm, typically without evident clinical symptoms. Prolonged displacement can lead to an increase in the carrying angle and significant upward movement of the radius, exceeding 7 mm, which may predispose to distal radioulnar joint dislocation (82). In order to ensure satisfactory reduction, all dysplastic changes and deformities must be corrected. The severity of observed dysplasia during open reduction directly impacts the difficulty in achieving radial head reduction and establishing a smooth rotational arc during reconstruction (Table 1).

**Table 1:** Studies treat neglected Monteggia fractures.

Time	Author	Treatment	Number of cases
2019	Soni, J.F (5)	Transverse osteotomy of the proximal ulna; and molded straight plate fixation of the ulna	6
2012	Armas (6)	Incisional reduction with wire fixation, and plate fixation were used, respectively and closed-replacement external fixation	35
2011	Rahbek, O (10)	Incisional repositioning and ulnar osteotomy	16
2022	Maszke, M (19)	Closed reduction and cast immobilization, Surgical treatment with internal fixation of the ulna	15
2017	Xu, Z (25)	Cut-and-replace combined with cora-based ulnar osteotomy	5
2007	Konrad, G.G (42)	Ulnar plate osteosynthesis with tension band wire fixation, radial head dislocation treated with closed reduction or cut-and-replace internal fixation. Ulnar plate placement with tension screws or stabilization with independent tension screws after indirect reduction of the fracture for comparison of efficacy	47
2007	Galik, K (46)	Radial head fracture fragments less than 1/3, fragments were excised. Radial head fractures fixed with headless screws, and radial head replacement	18
2019	Langenberg, L.C (71)	Corrective proximal ulna osteotomy with rigid plate fixation combined with annular ligament reconstruction	10
2017	Hoon, P (73)	Incision and repositioning alone, incision and repositioning plus ulnar osteotomy.	22
2018	Chen, H.Y (78)	Cutting and repositioning of the radial head and angulated osteotomy of the ulna extension	20
2019	Klug, A (81)	Surgical treatment consists of screw fixation of all type II and reconstructable type III fractures and, if reconstruction is not possible, radial head exchange (RHA) or surgical removal	78

However, the interval between traumatic dislocation and reconstructive surgery can influence surgical outcomes due to developmental abnormalities that may occur over time. Predicting the outcome of radial head resection in children is challenging; therefore, caution should be exercised when determining specific indications for treatment.

## 7 Conclusions

The diagnosis and treatment of neglected Monteggia fractures have undergone significant advancements over the past few decades. The distinctive injury pattern observed in pediatric cases necessitates careful consideration in all aspects of diagnosis and treatment. Physicians should meticulously assess physical examination findings, particularly focusing on radial head dislocation and brachioradial joint alignment on plain radiographs. Preoperative MRI also plays a crucial role in evaluating the condition of the radial head and radial notch. Neglected Monteggia fractures can often be misdiagnosed as simple ulna fractures until complications such as angular deformity of the forearm, bony proptosis, limited elbow motion with pain, neurological issues, or osteoarthritis changes arise. Although rare in children, prompt management is essential once identified. Currently, there is a consensus that neglected Monteggia fractures should be treated through ulna osteotomy with lengthening and angulation of ulna. However, further clinical data are needed to guide decisions regarding annular ligament reconstruction, selection of ulna osteotomy site, utilization of radial side humerus bone nail fixation, and surgical approach options. In the future, personalized surgical approaches for neglected Monteggia fractures in children could potentially benefit from one-to-one 3D reconstruction based on computed tomography (CT) scans. This technique allows accurate preoperative planning by combining CT-based 3D modeling with 3D printing for customized fixation methods and osteotomies (83). Such an approach has gained increasing popularity within orthopedics for precise correction of complex forearm deformities in pediatric patients.
